# Progress in Medicine

**Published:** 1895-12-07

**Authors:** 


### PROGRESS IN MEDICINE.

### FOODS AND NUTRITION.

**The Alcohol Question.**—A. R. Ledoux,[1] after a series of analyses, came to the conclusion that the adulteration of spirits and cigarettes is unimportant compared to the effect of the alcohol and nicotine they contain. The Committee of the British Medical Association[2] have issued a notice again explaining that the collective investigation report in no way shows that abstainers have shorter lives than moderate drinkers, for teetotalism being comparatively new, the average age of teetotalers must be less than that of the rest of the community, while anything beyond the most moderate drinking undoubtedly tends to shorten life. The consumption of beer in Great Britain has fallen from thirty-four gallons per head in 1874 to twenty-nine gallons in 1893; that of wine and spirits has risen from 1·30 gallons to 1·35; while tea and tobacco have increased largely.[3] A. L. Gillespie[4] read a statistical account of the patients at the Edinburgh Infirmary for five years who suffered from recent alcoholic disorders. They numbered 1,264, and the mortality was 3·4. The chief months were January and July, and the affections comprised delirium tremens, neuritis, pneumonia, and dyspepsia. The number from the professional classes was remarkable. A Japanese chemist, Mr. Takamine, has discovered a fungus by which starch can be converted into maltose without the ordinary process of malting the grain, and which also affords a yeast for bread-making or brewing. The diastase is of extraordinary activity, and the ferment more reliable and powerful than ordinary yeast.[5] The discovery of this aspergillus will probably cause a revolution in brewing and baking. In the treatment of dipsomania Skwozow reports remarkable success from large doses of strophanthus,[6] while in morphinomania Voison has always ordered sudden suppression of the drug, giving only a little alcohol.[7] He has never met with bad results from this plan, which, as medical officer to the Paris police, he has had many opportunities of testing.

**Adulterated and Diseased Foods.**—Among the accidents from ptomaines we notice that poisoning by unwholesome meat occurred lately, in Antrim from veal, at Leicester from tinned food, and in three instances in France from veal. It is stated that in most of the outbreaks from meat the animals have previously suffered from a special form of enteritis.[8] Seventy people were poisoned, with four deaths, at Limerick after eating a custard made with stale eggs and milk.[9] In other milk epidemics a bacillus like the B. coli was found in the milk.[10] Seven persons were poisoned by soup at Wolverhampton in August, and one died.[11] Aldridge reported instances from India, when the contaminated sand of the filters appeared to be the cause of outbreaks of fever and cholera.[12] The Lucknow epidemic was one of these. Thus the common filter may easily increase instead of preventing disease. The *British Medical Journal* draws attention to the report of its inquiry[13] as to the milk supply of London. Of the thirty samples only six were reported as genuine, and ten of the sophisticated also contained boric acid. Of the condensed milk, out of seventeen brands it was found that fourteen were made from skimmed milk, showing an average of 0·72 per cent. of fat instead of 10 to 12 per cent. The want of cleanliness was shown by the number of micro-organisms found in the "fresh" milk—500,000 per cubic cm., instead of about 30,000. The most frequent form detected was the Bacillus coli, possibly of fæcal origin, and the oïdium albicans was also found with others. The toxicity of borax when long continued is pointed out by Féré,[14] who finds that gastro intestinal symptoms are the first to appear, followed by affections of the skin, and albuminuria. With regard to the adulteration of othe foods, the *Lancet* points out that the frauds are now chiefly carried on by the wholesale[14] dealer, whom the Food and Drugs Act does not reach. Cocoa, cheese, butter, and spices are largely falsified, and 70,000 pounds of pepper containing only 1 per cent. of pepper have been openly sold at one time. A reform of the law is urgently demanded by the better class of dealers and others. The yeasts used in baking are of great importance.

**Bread.**—Vienna bread is made[15] with plain compressed yeast, English bread chiefly with a yeast grown in a mixture of malt and hops, Scotch with a flour-grown yeast, "leavened" bread with dough held over from a previous baking, which causes peptonisation and not alcoholic fermentation. In the Hovis flour the germs are first destroyed by superheated steam, as they otherwise keep badly; and in the London aerated bread a stale malt and flour yeast is used as well as carbonic acid pumped into the dough. A bread made from wood is largely employed in feeding the Berlin tram-horses.

**Hypodermic Feeding.**—The possibility of giving food by injections under the skin, when feeding by the mouth or rectum fails, has been attained, but there are narrow limits to its use.[16] Carbohydrates are painful, and peptones or albuminoses rapidly produce nephritis; however, fats such as camphorated oil can be injected even up to three ounces at a time, and are readily assimilated. Dogs can be even fattened in this way.

**The Digestion of Sugars.**—W. G. A. Robertson has published[17] some elaborate investigations on this point, and finds that the saliva has no effect in changing cane sugar into invertin; the gastric juice has some small power of doing so, which is connected with the acid present in it. The greater part, however, is changed in the small intestine by a special ferment. Now it has been thought that in gastric catarrh inversion proceeds more rapidly than in health, but this is entirely disproved, and absorption, too, is rendered much slower even in the case of inverted sugar. The latter causes much less pain and flatulence in dyspepsia than ordinary cane sugar; in other words, it is far more easily borne. This absence of discomfort was also noticed in other diseases, such as pernicious anæmia. Hence the dietetic value of dextrose and lævulose which together make up invert sugar. G. H. Bartley[18] has, however, attacked the use of glucose, which is an impure invert sugar, as actually causing dyspepsia and hindering the diastatic action of the saliva. Robertson's careful experiments seem to negative the view that the fermentative disturbances could be due to pure dextrose, and even Bartley's clinical arguments have been warmly opposed. The old question whether carbohydrates, fats, or proteids are the source of muscular energy is still the subject of experiments. Even Fick's noted results are shown to be uncertain, and D. Noel Paton and others have used improved methods, and conclude that[19] the body can utilise any of the three forms of food as sources of energy. Still, there must be sufficient proteid given to repair the machinery and to supply material for its growth. Hence the value of proteids in training for athletics as favouring the growth of energy-liberating muscles. Bouchard[20] takes a similar view, and insists on a double source of energy, which he calls the respiratory and nutritive mutations in the body, the one representing the circulating material, and the other the organised tissues, the former chiefly of carbohydrate origin, and the latter of proteid, but either are utilised when necessary. Chittenden's[21] elaborate review of digestive proteolysis shows, *inter alia*, that urea is formed chiefly from changes in proteid tissues, but also from excess of proteid food broken down into leucine. A product formed in the latter process, an immediate forerunner of urea, is now described as lysatine. From it about one-ninth of the urea is derived. The production of leucine and tyrosine is not apparently due to any micro-organism in the intestines, but only to the pancreatric ferment. Vincent D. Harris recently cultivated some twenty varieties of these microbes, and reported that they had very little to do in the formation of leucine, in the inversion of sugar, or conversion of starch; and, in short, that their *rôle* was less important than had been thought.[22] On the other hand, Vaughan Harle[33] found that in obstruction of the pancreatic duct there was not only a lessened absorption of fa's, but also of proteids.

[1] Med. Rec., March 12. [2] B.M.J., Dec. 8. [3] Med. Rec., April 20. [4] Ed. Med. J., Sept. [5] Lanc., May 25. [6] Pract., June. [7] Med. Week, June 29. [8] B.M.J., June 29. [9] Lanc., July 20. [10] Med. Chron., Feb. [11] Lanc., Sept. 21. [12] Lanc., Feb. 16. [13] B.M.J., July 13, Aug. 3. [14] Int. Med. Mag., May. [15] Lanc., Sept., 21. [16] Ch. and Druggist, Aug. 17. [17] Med. Week, April 19. [18] Edin. Med. J., Dec., 1894. [19] N.Y. Med. J., March 20. [20] Ed. Med. J., June, 1895. [21] Med. Week. June 8. [22] B.M.J., April 27. [23] Path. J. and Bacteriology, July. [24] Ibid.

